# Effect of hydroalcoholic seed extract of *Nigella sativa* on hepatic and pancreatic factors of Nrf2 and FGF21 in the regulation of insulin transcription factors of MafA and PDX-1 in streptozotocin-treated diabetic rats

**DOI:** 10.1186/s12986-022-00699-9

**Published:** 2022-09-15

**Authors:** Mahsa Soleimani-Dodran, Reza Alipanah-Moghadam, Farhad Jeddi, Mohammad Babaei, Ramin Salimnejad, Elham Bahreini

**Affiliations:** 1 Department of Biochemistry, Faculty of Medicine, Iran University of Medical Sciences, Tehran, Iran; 2 Department of Clinical Biochemistry, School of Medicine, Ardabil University of Medical Sciences, Ardabil, Iran; 3 Department of Genetics and Pathology, School of Medicine, Ardabil University of Medical Sciences, Ardabil, Iran; 4 Department of Clinical Sciences, Faculty of Veterinary Science, Bu-Ali Sina University, Hamedan, Iran; 5 Department of Anatomical Sciences and Pathology, School of Medicine, Ardabil University of Medical Sciences, Ardabil, Iran; 6 Department of Biochemistry, Faculty of Medicine, Iran University of Medical Sciences, Tehran, Iran

**Keywords:** Diabetic, Insulin, *Nigella sativa*, FGF21, NRF2, PDX1, MafA

## Abstract

**Introduction:**

*Nigella sativa* (*N. sativa*), one of the most commonly used medicinal herbs with antioxidant properties, increases blood insulin levels and lowers fasting blood sugar. Nuclear Erythroid Factor-Related Factor 2 (Nrf2) and Fibroblast Growth Factor 21 (FGF21) are two antioxidant factors that are increased by oxidative stress and hyperglycemia. The present study investigated how hydroalcoholic extract of *N. sativa* seed (HENS) increases blood insulin levels, taking into account changes in antioxidant factors and expression of insulin transcription factors.

**Materials and methods:**

Two groups of male diabetic wistar rats were treated orally with HESN at doses of 200 and 400 mg/kg-body weight for one month. Fasting blood sugar (FBS) and insulin were measured using standard kits by photometric and ELISA methods, respectively. The expression levels of the Nrf2, FGF21 and β-Klotho genes as well as the insulin gene-stimulating transcription factors of MafA and PDX-1 were evaluated using real-time PCR. Oxidative stress was assessed by assessing serum total oxidation status (TOS), malondialdehyde (MDA), and total antioxidant capacity (TAC).

**Results:**

HSEN showed a significant reducing effect on FBS and oxidative biomarkers and an increasing effect on serum insulin levels in treated diabetic rats compared to untreated diabetics (*P* < 0.05). The elevated levels of NRF2 and FGF21 in the liver and pancreas of the diabetic control group were significantly reduced after treatment with both HESN doses (*P* < 0.05). Following the ameliorative effects of HENS on pancreatic tissue and the reduction of oxidative stress, the expression level of MafA and PDX1 genes approached the level of these factors in healthy rats (*P* < 0.05).

**Conclusion:**

This study showed the therapeutic effects of HENS on diabetic pancreas by reducing oxidative stress and tissue damage, modifying the expression levels of PDX-1 and MafA genes, and regulating insulin secretion and blood glucose levels.

## Introduction

Diabetes is one of the world's greatest healthcare challenges. It is a clinical disorder associated with metabolic abnormalities such as hyperglycemia, hyperlipidemia, and inflammation that result in the formation of reactive oxygen/nitrogen species (RONS) [1, 2]. The main cause of complications in diabetes, including micro- and macrovascular complications of nephropathy, retinopathy, cardiomyopathy and other complications caused by oxidative stress resulting from high production of RONS [3]. Nuclear erythroid factor 2-related factor 2 (Nrf2) is a transcription factor whose increase in response to oxidative stress has been confirmed by several studies. It produces proteins that respond to environmental stress, cell damage, and inflammation, and protect tissues from free radicals and oxidative stress. The Nrf2 signaling pathway is one of the most important cellular defense mechanisms against oxidative stress [4, 5]. Several cell and animal studies have reported increased Nrf2 expression and its protective effects against hyperglycemia-induced oxidative stress [6, 7]. Compared to other tissues, β-cells express low levels of antioxidants such as superoxide dismutase and catalase, while expression of other antioxidants such as thioredoxin reductase and glutathione peroxidase, which are Nrf2 target genes, is significant in β-cells [8, 9]. Besides preventing oxidative stress, Nrf2 plays a role in metabolic homeostasis, including lipid metabolism and energy expenditure, and suppresses gluconeogenesis; so that Nrf2-knockout rats show insulin resistance and weight loss [10].

Fibroblast growth factor 21 (FGF21), which plays an important role in glucose and lipid metabolism, is one of target genes of Nrf2 [11, 12]. Circulating levels of FGF21, primarily derived from the liver, are elevated in metabolic disorders such as obesity, diabetes and cardiovascular disease to help maintain energy homeostasis [13]. It regulates energetic hemostasis by increasing glucose uptake in adipose tissue, increasing lipolysis and increasing production of ketone bodies by the liver. Studies have shown that FGF21 has a protective effect on β-cell function in the pancreas, such that its removal leads to β-cell failure and suppression of insulin secretion [12, 14]. Studies have shown that administration of FGF21 in a diabetic model reduces insulin resistance and reduces hyperglycemia by increasing the expression of transcription factors that stimulate the insulin gene [13–16]. In addition, FGF21 plays a potential role in increasing adiponectin expression [17]. Adiponectin, secreted from the adipose tissue, enhances insulin secretion through the upregulation of transcription factors regulating insulin gene expression including MafA and PDX1 [18]. PDX1, an important factor in the differentiation of pancreatic endocrine cells into β-cells, is produced early in pancreatic development, while MafA, which is specific for β-cells, is expressed early in the secretory phase [19, 20]. Both factors are potential activators of insulin gene transcription. Previous studies have shown that oxidative stress lowers levels of the factors MafA and PDX1, leading to insulin resistance [19, 21].

*Nigella sativa* (*N. sativa*) from the Ranunculaceae family, widely used in Traditional Iranian Medicine (ITM), is one of the medicinal herbs effective in lowering blood sugar and lipid profile in diabetics. This plant is rich in polyphenolic compounds such as thymoquinone and flavonoids, which may play a potential role in cell protection and repair by increasing antioxidant capacity and reducing oxidative stress [22, 23]. The effects of *N. sativa* seed extract on increasing blood insulin and lowering blood sugar levels have been demonstrated in the previous studies [22, 24].

Oxidative stress can modulate a wide variety of biological processes by inducing known and unknown signals that modify gene expression. Transcription factors are the principal nuclear factors that control the expression of other genes. Therefore, two groups of genes were considered in this study, one group is transcription factors that are activated by oxidative stress and have soothing effects on damaged tissue including Nrf2, FGF21, and β-kloto, and the other group is transcription factors related to insulin gene stimulation including PDX1 and MafA, whose expression may be affected by the transcription factors of the first group. Due to insufficient information about the mechanism of *N. Sativa* in improving insulin secretion in diabetes, the aim of this study was to investigate the relationship between the antioxidant effects of *N. sativa* and the expression levels of MafA and PDX-1, as two important transcription factors involved in insulin secretion. Most studies have reported reduced expression of MafA and PDX-1 in type 2 diabetes [22, 24], but no study was found to clarify the effects of *N. sativa* on MafA and PDX-1 changes in STZ-induced diabetes model (type 1 diabetes).

## Material and methods

### Preparation of hydro alcoholic extract of *N. sativa*

*Nigella sativa* seed was purchased from a herbal store. After evaluation and approval by the Herbarium Center for Medicinal Plants of Tehran Medical University, herbbarcode DMP-846 was assigned. The hydroalcoholic extract was obtained with certain modifications using the maceration method [25]. Briefly, 1 kg of *N. sativa* seed was ground and soaked in 4 l of 70% ethanol for 72 h in the dark place at 40 °C. The prepared extract was concentrated, dried in a rotary evaporator at 50 °C and stored refrigerated at 4 °C.

### Animal studies

The study was approved by the Ethics Committee of Iran University of Medical Sciences (IR.IUMS.REC.1399.1341). Twenty-four male Wistar rats with an average weight of 220 ± 20 g and mean blood glucose of 85 ± 6.6 mg/dl kept at the experimental research center of the university for 4 weeks. After separating of six rats as a negative or healthy control group, the remaining rats received streptozotocin (STZ) intraperitoneally at a single dose of 60 mg/kg. After 3 days, the rats with a blood sugar level above 250 mg/dl were considered diabetic. Diabetic rats were separated into three groups: diabetic control group (DC), the group treated with 200 mg/kg HENS (E-200), and the group treated with 400 mg/kg HENS (E-400).

HENS was administered orally for 4 weeks. After an overnight fast of 10 to 12 h, the rats were first anesthetized, and blood was drawn directly from the heart. The liver and pancreas were then immediately removed, washed with saline, placed in liquid nitrogen and stored at − 70 °C until RNA extraction. Whole blood was centrifuged at 4500 rpm for 20 min and isolated sera were stored at − 70 °C until analysis.

### Biochemical experiments

#### Fasting blood sugar (FBS) and serum insulin assay

Serum glucose levels were measured using a standard kit using the photometric method. Serum insulin levels were measured using ELISA RayBio® Rat Insulin (MBS724709; MyBioSource Company) according to the manufacturer's instructions. Homeostasis model assessment tests (HOMA), including insulin resistance (HOMA-IR) and Quantitative Insulin Sensitivity Check Index (QUICKI) were calculated as follows [26, 27]:

$$\begin{aligned} {\text{HOMA - IR}} & = {\text{ Insulin }}\left( {\upmu {\text{U/ml}}} \right) \, \times {\text{ glucose }}\left( {\text{nmol/L}} \right)/22.5 \\ {\text{QUICKI}} & = = 1/ \, \left[ {\log \left( {{\text{insulin }}\,{\text{in}}\,\upmu {\text{U/ml}}} \right) + \log \left( {{\text{glucose}}\,{\text{ in }}\,{\text{mg/dl}}} \right)} \right]. \\ \end{aligned}$$

#### Evaluation of oxidative stress indices

STZ is highly cytotoxic to pancreatic beta cells through induction of reactive oxygen/nitrogen species (ROS/RNS) formation, and metabolic complications. The protective effect of *N. sativa* on the pancreas was evaluated by assessing the status of oxidative stress profiles and histological changes (refer to Histopathology part).

- Total antioxidant levels: Total serum antioxidant status (TAS) was measured using iron-reducing antioxidant potency [28] according to the manufacturer's instructions. The reduction of Fe^3 +^ to Fe^2 +^ by the sample was taken as an indicator of antioxidant power. In this method, the complex between Fe^2 +^ and tripyridyltriazine (Fe^2+^-TPTZ) produces a blue color with maximum light absorption at 593 nm. The results were compared to the standard curve obtained from a serial dilution of Fe_2_SO_4_ (range from 100 to 800 μMol) in 1 ml of FRAP reagent (300 mM acetate buffer, 10 mM TPTZ/HCl solution and 20 mM ferric chloride). The values were expressed as µM Fe^2+^.
- Total oxidant levels: ROS generation was measured by a fluorometric assay using 2,7-dichlorofluorescein diacetate (H_2_DCF-DA) [29]. In this assay, H_2_DCF-DA is deacetylated to H2DCF by endogenous serum esterases and then oxidized to dichlorofluorescein (DCF) by ROS types. DCF fluorescence was measured at 485 nm excitation and 528 nm emission using a Synergy HT microplate reader (BioTek Instruments) set at 37 °C. H2DCF is essentially non-fluorescent but becomes highly fluorescent when converted to DCF by the presence of ROS such as superoxide anion radicals (O^2−^), hydroxyl radicals (OH^−^), hydrogen peroxide (H_2_O_2_), hypochlorous acid (HOCl), peroxyl (RO^2−^), Alkoxyl (RO^.^), hydroperoxyl (HO^2−^), singlet oxygen (1O_2_) and peroxynitrite (ONOO-).
- Determination of lipid peroxidation: The lipid peroxidation (LPO) assay was based on the ability of malondialdehyde (MDA) to conjugate with 2-thiobarbituric acid (TBA) to form a pink product with a maximum absorbance at 532 nm [30]. Briefly, 200 µl of each sample was mixed with 20% trichloroacetic acid (TCA) and the generated precipitate was dispersed in H_2_SO_4_ (0.05 M). After addition of thiobarbituric acid (TBA) (0.2% in sodium sulfate), the sample was heated in a boiling water bath for 30 min. The LPO adducts were then extracted with n-butanol and the absorbance was measured at a wavelength of 535 nm. The MDA content was expressed in nmol/ml.

### Assessing gene expression

#### Total RNA extraction, cDNA synthesis and quantitative real-time PCR (RT-PCR)

Investigation of gene expression levels of Nrf2 and FGF21 in both liver and pancreas and of β-Kloto, PDX1 and MafA in pancreas was performed using real-time quantitative PCR (RT-qPCR) technique. Each tissue sample was pulverized by placing in a mortar containing liquid nitrogen, then total RNA extraction and purification was performed using TriPure Isolation Reagent (11667157001, Sigma, UK) according to kit instructions carried out. Then the quality of the RNA was assessed by electrophoresis and the purification and concentration of the RNA using the NanoDrop instrument (BOE 8637000, Micro UV/Vis spectrophotometer model N − 1, Hamburg, Germany). Total RNA was reverse transcribed into cDNA using the qPCRBIO cDNA synthesis kit (PB30.11-10, PCR Biosystems, UK) according to the manufacturer's instructions.

The GAPDH gene (glyceraldehyde-3-phosphate dehydrogenase) was considered as an internal control to quantify the expression of the target genes. The synthesized cDNA was subjected to RT-qPCR quantification using Super SYBR Green qPCR Master Mix (cat.# YT2552, Yektatajhize, Iran) and appropriate forward and reverse primers developed by NCBI primer design were (Table 1). The amplification cycle threshold (Ct) was determined and normalized by the GAPDH value. The fold change in expression was calculated using the 2^−ΔΔCt^ method. Table 1 presents the real-time PCR primer sequences and annealing temperatures.

**Table 1:** Primer sequences and annealing temperatures

Gene		Sequence	Tm-annealing °C
NRF2	F	TGTGGGGTAAGTCGAGAAGTG	58
	R	TACTGGGCTGGCTGAATTGG	
FGF21	F	TTCGGGACTGTGGGTCTGTCTC	61.5
	R	TTTGCAGGTGGGCTTCGGTG	
MAFA	F	TCGTCAACGACTTCGACCTG	58
	R	TCAGAAGCTGGGCGAAGAG	
PDX1	F	TTCCGGACCTTTCCCGAATG	59
	R	TTCTGCTGCGTATGCACCTC	
Β-KLotho	F	TGCATTGGTTCTGCTGCGAG	59
	R	TAAATGCTCCGGTCCCAACG	
GAPDH	F	TCATCAACGGCACAGTCAAGG	60
	R	TTCTGCATGGTGGTGAAGACG	

### Histopathology

After fixation in 10% neutral buffered formalin, tissue was dehydrated in increasing concentrations of ethanol, cleared in xylene, then infiltrated and embedded in paraffin. Paraffin-embedded tissue was sectioned at 5–6 µm using a rotary microtome (Leica RM2255, Germany), then the sections were mounted on glass slides. After deparaffinization, slides were stained with hematoxylin and eosin (H&E). All slides were examined by a veterinary pathologist using a light microscope (Olympus CX41, Japan) equipped with a digital camera (Olympus DP25, Germany) at 40 × and 200 × magnification.

### Statistical analysis

Statistical analysis was performed using SPSS version 22 software and GraphPad Prism9. Data normality was verified using the Shapiro–Wilk normality test. Differences between individual groups were determined by one-way ANOVA analysis followed by Tukey's test for pairwise comparisons. Values ​​were expressed as means ± SD. A *p*-value < 0.05 was considered statistically significant.

## Results

### Effects of HENS on insulin and blood sugar

The effect of HENS on insulin production was evaluated by measuring blood insulin levels and comparing the data to blood glucose levels, HOMA indices and insulin-stimulating transcription factors.

**Fig. 1:**
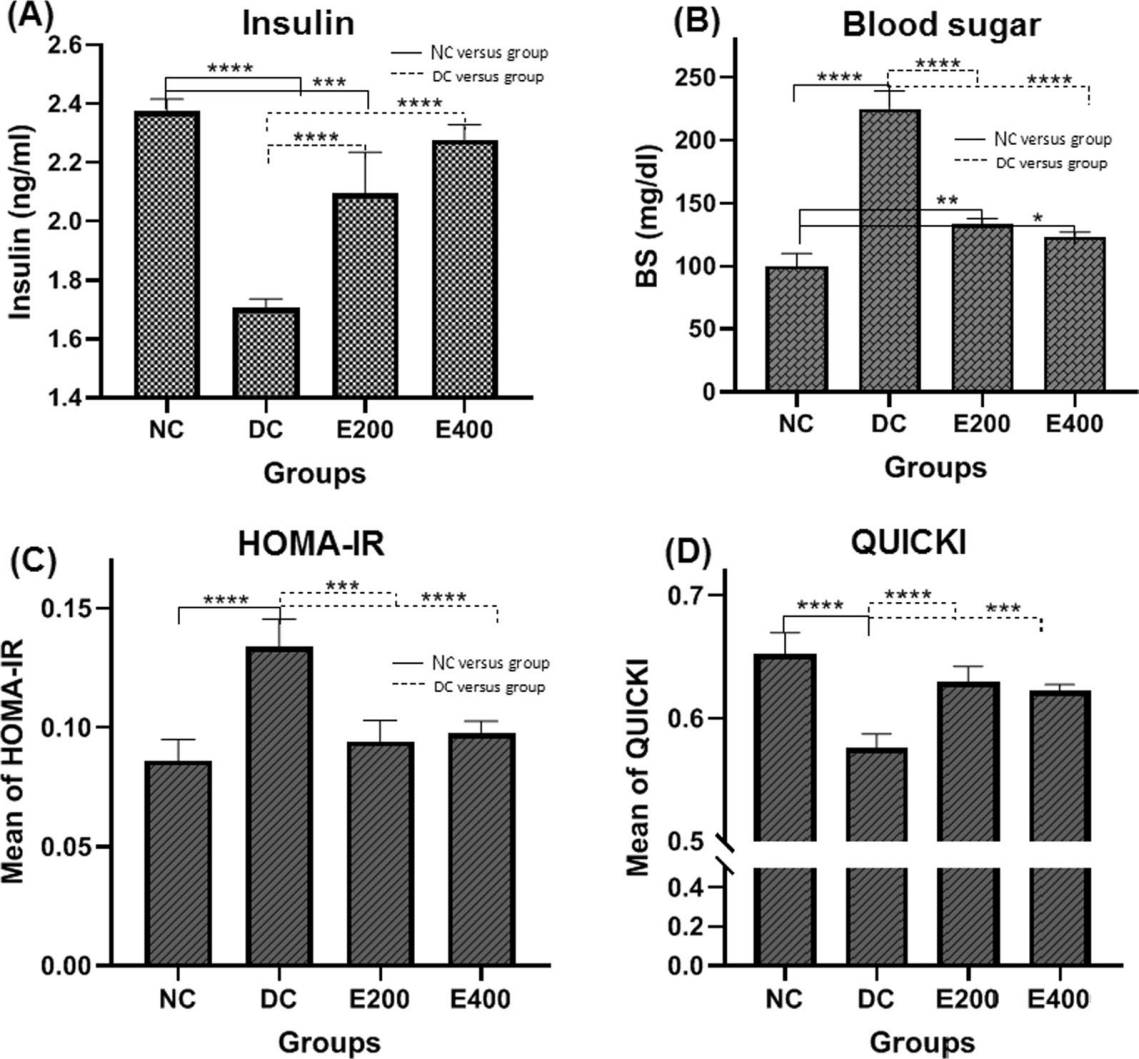
Effects of HENS on biochemical parameters  including: insulin (**A**), blood sugar (**B**), HOMA-IR (**C**) and QUICKI (**D**). Values are presented as means ± SD. HOMA-IR: homeostatic model assessment-insulin resistance, *QUICKI*: quantitative insulin sensitivity check index, *NC:* normal control, *DC:* Diabetic control, HENS: hydroalcoholic extract of *N. sativa* seeds. *P-value obtained using one-way ANOVA

Figure 1 shows the dose-dependent effect of HENS on insulin (Fig. 1-A), blood glucose (Fig. 1-B), and HOMA indexes of HOMA-IR (Fig. 1-C) and QUICKI (Fig. 1-D) which were measured at the end of the expriment. One-way ANOVA showed a significant difference in insulin concentration (*P* < 0.001) and also in blood glucose (*P* < 0.001) between the groups. Blood insulin levels were significantly higher in both HENS-treated groups than in the diabetic control group (*P* < 0.05). Despite the non-significant difference in blood insulin levels between the E-400 group and healthy controls (*P* > 0.05), a significant difference in blood glucose concentration (*P* < 0.05) was observed.

**Fig. 2:**
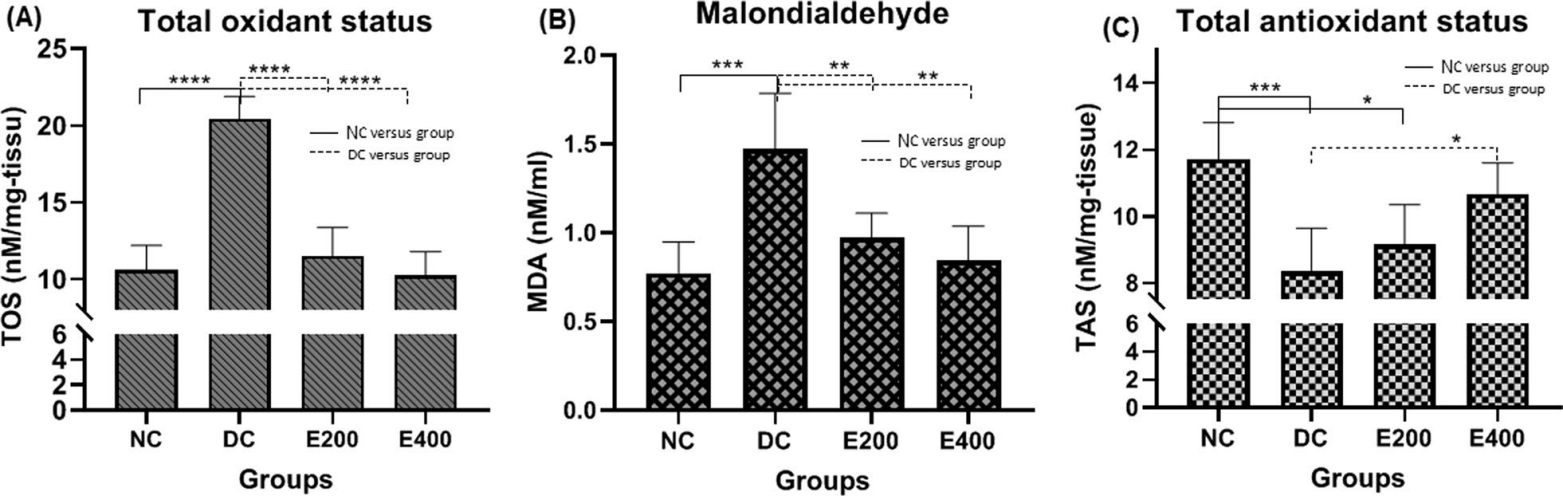
Comparison of the effect of HENS on total oxidant status (TOS) (**A**), Malondialdehyde (MDA) (**B**), and total antioxidant status (TAS) (**C**) in the study groups

HOMA-IR and QUICKI significantly were increased and decreased, respectively in DC group compared to NC group (*P* < 0.001 for each variable). HENS administration reversed the process in diabetic rats to degree that significantly differed from the DC group (*P* < 0.001 for two doses of HENS). Figure 2C and D respectively, indicate pair-group comparison for HOMA-IR and QUICKI.

### The effect of HENS on oxidative stress

Figure 2 compares serum levels of oxidative stress markers including total oxidant (TOS), total antioxidant (TAS), and MDA, in HENS-treated groups versus healthy controls and diabetics. As expected, serum levels increased compared to the healthy group oxidative stress parameters (TOS and MDA) and total antioxidant levels decreased in the diabetic control group (*P* < 0.001). HENS treatment decreased TOS and MDA levels and increased TAS levels in a dose-dependent manner, such that MDA and TAS levels in the E-400 group were compatible with healthy control (*P* > 0.05).

### The effect of HENS on the gene expression levels of Nrf2, FGF21 and β-Kloto

**Fig. 3:**
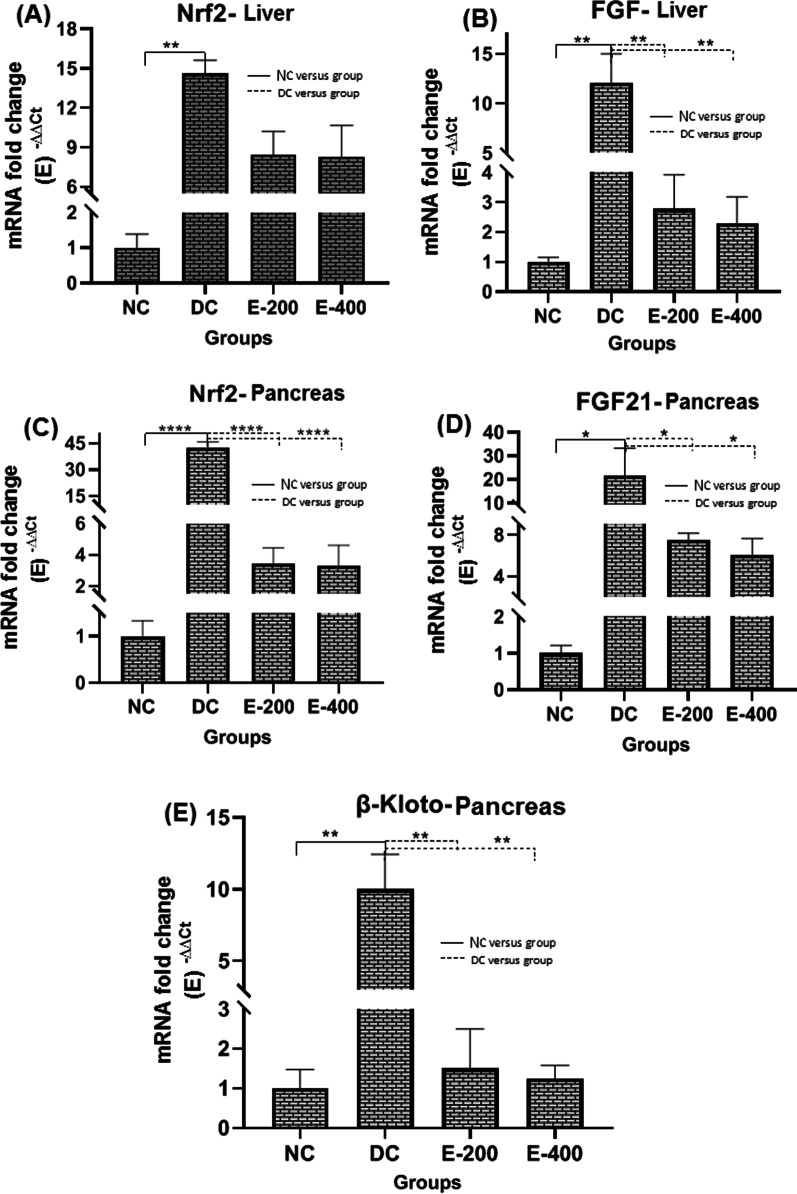
Comparison of mRNA fold changes in Nrf2-liver (**A**), FGF21-liver (**B**), Nrf2-pancreas (**C**), FGF-pancreas (**D**) and β-Kloto-pancreas (**E**) gene expression levels in the study groups.

Figure 3 shows levels of gene expression of Nrf2 and FGF21 in liver and pancreas. Although the aim of the study was to investigate the effects of HENS on changes in the levels of both factors related to insulin secretion in the pancreas, since the origin of FGF21 in plasma is mainly hepatic, the effects of HENS on the hepatic levels of both factors were also evaluated. Since the half-life of FGF21 is around 0.5 to 2 h, it was preferred to measure its gene expression level rather than measuring its blood level.

Compared to the healthy control group, the expression levels of Nrf2 and FGF21 genes were increased in the liver and pancreas of diabetic rats with higher expression levels in the pancreas. (*P* < 0.05). HENS treatment reduced the level of gene expression of these factors in the liver and pancreas of diabetic rats in a dose-dependent manner. Compared to healthy and diabetic controls, this decrease was not significant in the liver (*P* > 0.05), but in pancreatic tissue, this decrease was significantly different from the diabetic control group (*P* < 0.05), but comparable to the healthy control group (*P* > 0.05).

Changes in the expression level of the β-Kloto gene were consistent with those of FGF21. A significant increase in the expression level of β-Kloto gene in pancreas of DC group was observed compared with NC group (*P* < 0.001). HENS administration reduced β-Kloto in diabetic rats to levels comparable to those in the NC group (*P* > 0.05). However, these reductions were not dose-dependent.

### The effect of HENS on genes involved in insulin production

**Fig. 4:**
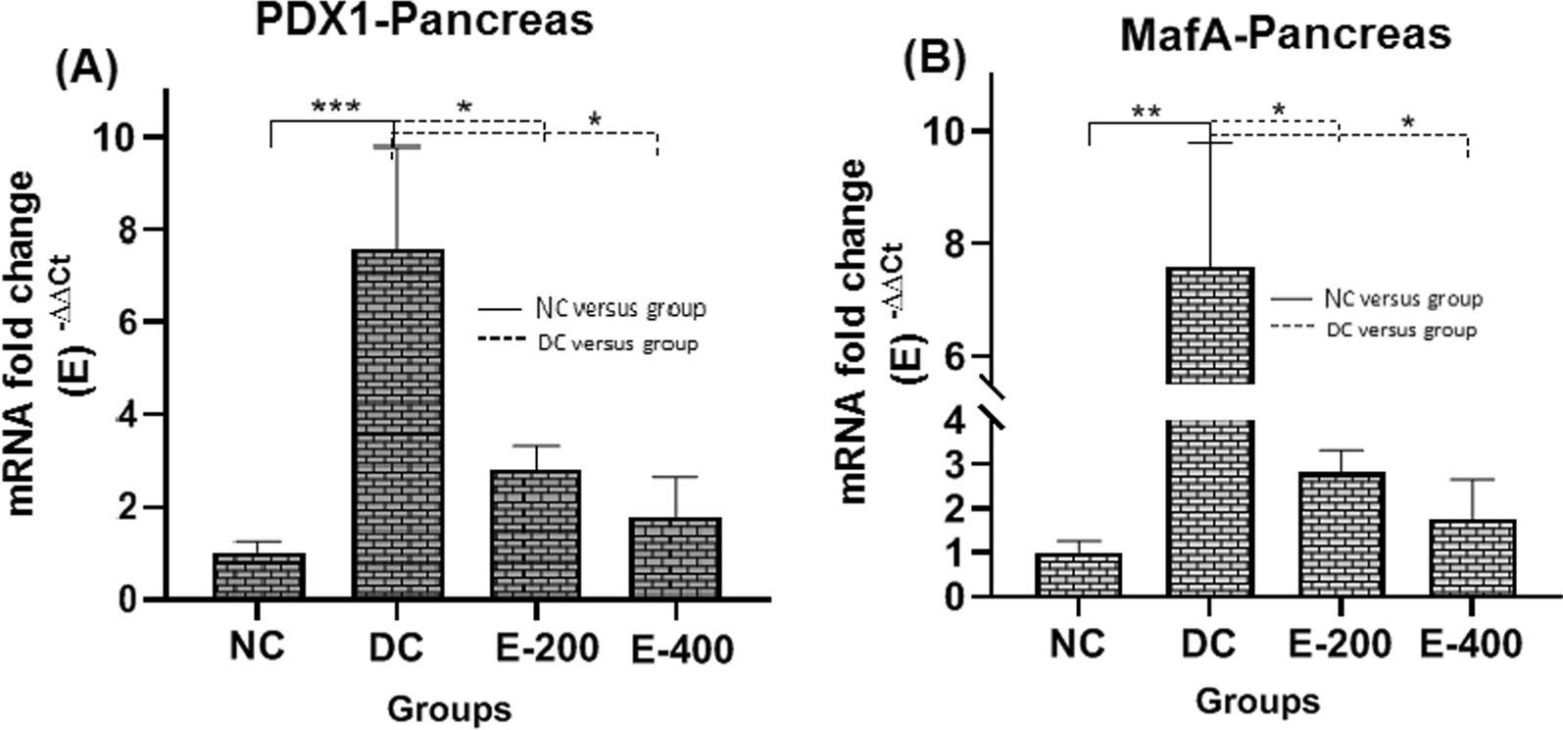
Comparison of mRNA fold changes in PDX1 (**A**) and MafA (**B**) gene expression levels in pancreatic tissue in the study groups

Compared with the NC group, a significant increase in the gene expression levels of PDX1 and MafA was observed in the pancreas of the DC group (*P* < 0.001). HENS administration significantly lowered PDX1 and MafA levels in a dose-dependent manner in diabetic rats (*P* < 0.05), so that the levels of PDX1 and MafA become non-significant in comparison with the NC group (*P* > 0.05) (Fig. 4).

### Effects of HENS on pancreatic histology

**Fig. 5:**
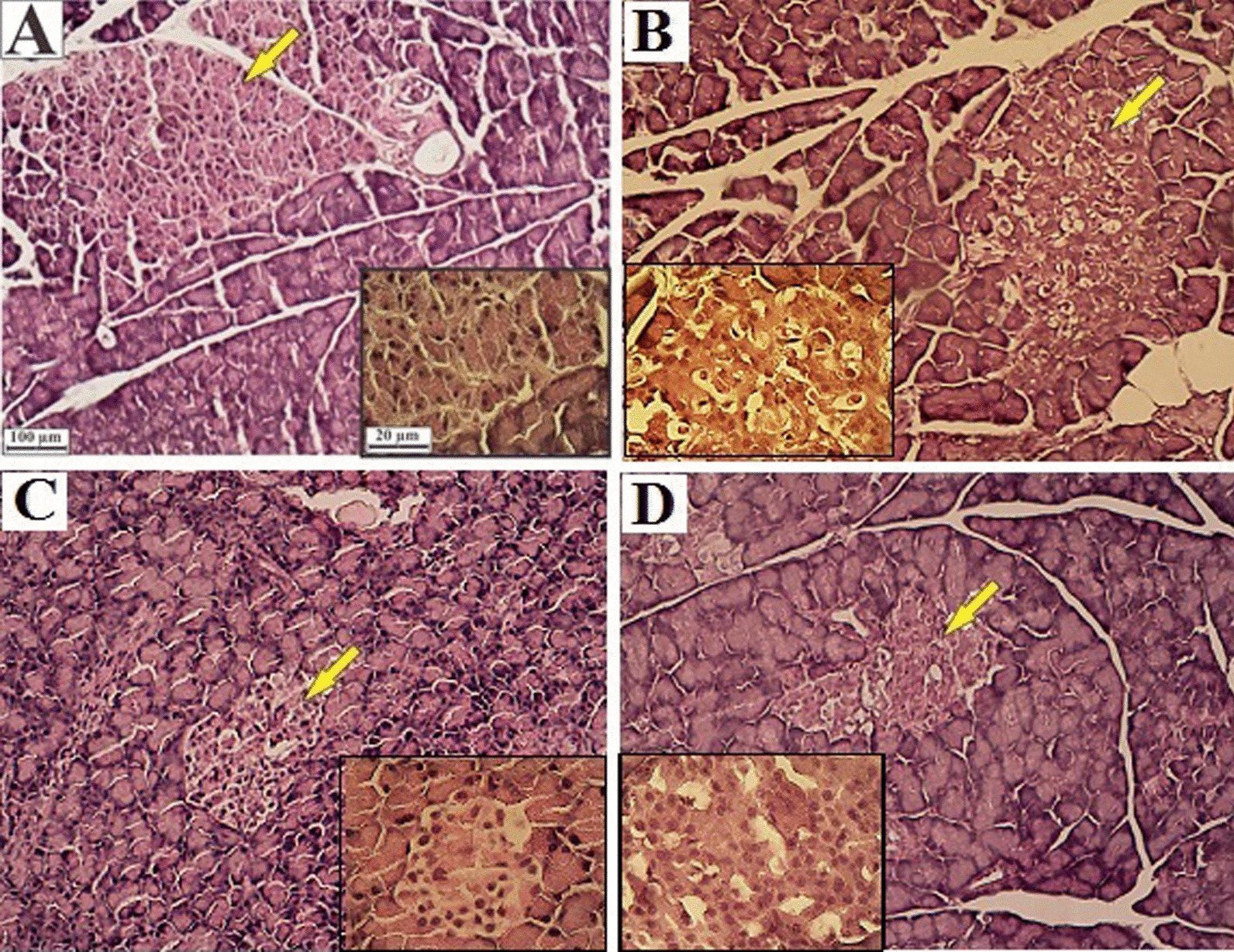
Morphological assessment of pancreatic tissue; H&E staining. Magnification × 100 and × 400. **A** Normal control group with the normal tissue structure; **B** Diabetic control group with vacuolated cells, inflamed appearance, and reduced cell density; **C** HENS-200 group with a slight decrease in cell density and inflammation in the islets of Langerhans; **D** HENS-400 group with normal cell size and histological structure

Histopathological examination of the pancreas showed normal cell size and healthy structure of the islets of Langerhans in the NC group (Fig. 5-A). But in the DC group (Fig. 5-B), a decrease in cell number, the presence of vacuolar cells and dystrophy of the islets of Langerhans were observed. The intensity of the mentioned symptoms was lower in the E-200 and E-400 groups than in the DC group (Fig. 5-C and D, respectively); such that the cell size and tissue structure of the pancreas in the E-400 group were similar to those in the NC group.

## Discussion

The effects of *N. sativa* seed extract on increasing blood insulin and reducing blood sugar levels has been demonstrated in the previous studies, so the purpose of this study was to clarify how *N. sativa* seed extract increases blood insulin [22, 24]. Due to the important role of hyperglycemia and consequent oxidative stress in the pathogenesis of diabetes, elucidating how *N. sativa* seed extract increases blood insulin was investigated by finding a relationship between the antioxidant effects of *N. sativa* seeds and factors involved in insulin gene expression. Therefore, two groups of transcription factors were considered: transcription factors activated by oxidative stress and having soothing effects on damaged tissues, and transcription factors related to insulin gene stimulation, whose expression is influenced by the transcription factors of the first group.

Our data indicated that HESN treatment increases blood insulin and reduces the blood sugar levels in diabetic rats which were consistent with previous studies [24, 31]. In addition, the DC group exhibited a state of tissue resistance to insulin (HOMA-IR) and reduced sensitivity to insulin (QUICKI). This is attributed to STZ injection-induced hyperglycemia causing progressive insulin resistance in peripheral tissues due to decreased serine/threonine kinase and/or tyrosine phosphatase activity. This reduction leads to decreased autophosphorylation of the insulin receptor and reduced translocation of Glut-4 to the plasma membrane [32, 33]. HENS treatment had improved effects on the HOMA indexes, which could be due to reduced insulin resistance in peripheral tissues, increased insulin secretion from the pancreas, or repair of damaged β-cells.

Another important feature of *N. sativa* seed is its antioxidant properties, which were characterized by a significant decrease in ROS and MDA, as well as a significant increase in FRAP levels in HENS-treated diabetic animals. The hypoglycemic and antioxidant effects of HENS may be attributed to the content of phenols and flavonoids, particularly thymoquinone, the active component of *N. sativa* [34]. Thymoquinone also reduces the expression of enzymes involved in hepatic gluconeogenesis and gut glucose uptake, which has been attributed to activation of AMPK in the liver and gut [35]. On the other hand, Abdelmeguid et al. [36], El-Shemi et al. [37], and Pelegrin et al. [38] attributed the hypoglycemic effects of *N. sativa* to the insulinotropic effects of thymoquinone, and reported that thymoquinone could regenerate pancreatic β-cells to secrete insulin. In the present study, the histopathological results of pancreatic tissue showed a reduction in inflammation and a partial improvement in islets after treatment of diabetic rats with both doses of the extract, particularly dose 400. Such improvement may be attributed to partial regeneration or repair of damaged β-cells as well as modulation of insulin synthesis in surviving β-cells, however, this assumption needs to be further investigated in the relevant cell line.

Acute hyperglycemia causes the MAPK, PKC and PI3K signaling pathways to be activated under conditions of acute hyperglycemia in β-cells [39, 40]. MAPK activation phosphorylate Clu3 and Keap1, releasing Nrf2 from the complex. Free Nrf2 counteracts oxidative stress by entering the cell nucleus and inducing the expression of endogenous antioxidant genes. Furusawa et al., showed that Nrf2 exerts some of its protective effects by inducing FGF21 expression [12]. In the present study, increased levels of Nrf2 and FGF21 expression in the liver and pancreas of diabetic rats were associated with increased body oxidative stress. Significant increase in the pancreatic levels of Nrf2 and FGF21 compared to their levels in the liver may be attributed to STZ-induced damage in the pancreas. Previous studies reported that FGF21 increases the function and survival of β cells, inhibits glucagon secretion, and prevents inflammation of the pancreas [16, 41]. But the histopathological results of the DC group showed tissue damage despite the high expression of Nrf2 and FGF21 in the pancreas. This discrepancy can be attributed to chronic hyperglycemia, a poor antioxidant system, and continuous β-cell production of ROS that suppresses the protective response. After HENS treatment and reduction in ROS levels, Nrf2 and FGF21 levels decreased in both pancreas and liver, but the decrease in Nrf in the pancreas was significantly greater than in the liver. The effects of FGF21 via the FGF21 receptor are mediated by the cofactor β-Klotho. A correlation between gene expression changes in β-Klotho and FGF21 was observed in the experimental groups, which could be due to the presence of a common stimulator for both, namely ATF4 [42].

Although there is no report on the direct effect of Nrf2 on insulin transcription factor levels, it indirectly has a positive effect on the synthesis and secretion of insulin by controlling the activity of the antioxidant system of pancreatic tissue and increasing the expression of FGF21. However, basal secretion of FGF21 in the healthy pancreas is necessary for survival and maintenance of β-cell function. Chen et al. reported that exogenous FGF21 increases insulin secretion in islets isolated from the pancreas of an FGF21 knockout model. Pan et al. observed decreased FGF21 expression as well as decreased insulin transcription factors in the db/db mouse model [16]. After treating the islets isolated from the pancreas of these mice with the viral vector carrying the FGF21 gene and inducing high expression of FGF21 in β cells, they observed an increase in the expression of insulin gene transcription factors. FGF21 is a stimulator of the PI3K/PKB signaling pathway [17]. As explained later, activation of PKB inactivates FOXO and stimulates insulin gene expression, but in chronic hyperglycemia and oxidative stress, this pathway is weakened in β-cells and PDX1 and then insulin gene expression is reduced.

Hyperglycemia and oxidative stress are two important factors in the development of diabetes and are associated with altered expression levels of insulin gene-stimulating transcription factors. Although some previous studies have reported decreased expression levels of MafA and PDX1 in type 2 diabetes [20, 43], the present study showed increased expression levels of both factors in STZ-diabetic rats. The difference between the two diabetic models is that, in type 2 diabetes, hyperglycemia occurs first and then oxidative stress, but in the STZ diabetic model, oxidative stress occurs first and then insulin depletion and hyperglycemia.

What is happened in type 2 diabetes: Iwaoka et al. showed that AMPK inhibition increases MafA protein accumulation as well as GLUT2 and insulin gene expression in β-cells [44]. AMPK acts as an energy sensor in the cell and is activated by a decrease in ATP and an increase in AMP [45]. Previous studies have shown that AMPK activity is suppressed in chronic hyperglycemia. FOXO1 is another transcription factor that protects β-cells from oxidative stress by complexing with the promyelocytic leukemia protein Pml and the NAD-dependent deacetylase Sirt1 to activate NeuroD and MafA expression [46] and inactivate PDX1. Under normal conditions, insulin released from secretory granules following a post-feeding rise in blood glucose affects β-cells in autocrine manner and activates the PI3K-PKB pathway [47]. PKB (or Akt) inhibits FOXO1 through phosphorylation [40]. Phosphorylated FOXO1 fails to enter the nucleus to reduce PDX1 gene expression and nuclear concentration. This activates the expression of the insulin gene. In persistent hyperglycemia, PI3K- PKB signaling activity is impaired due to phosphorylation of the IRS by PKC [48]. In addition, oxidative stress oxidizes PKB at cysteine ​​residues by H_2_O_2_. Because oxidized PKB cannot phosphorylate and inactivate FOXO1, so FOXO1 enters the cell nucleus and inactivates PDX1 by changing its phosphorylation pattern, moving from the nucleus to the cytoplasm and reducing its gene expression [49]. In the present study, the decrease in MafA and PDX1 levels in the pancreas of healthy fasted rats (NC group) can be attributed to low blood glucose and high AMP/ATP ratio in β-cells. But how are the high MafA and PDX1 levels to be interpreted in diabetic STZ rats (DC group)?

STZ enters β-cells via GLUT2 and induces toxicity by producing ROS/RNS. The weak antioxidant system of β-cells causes activation of the MAPK pathways, including kinase families of ERK, JNK and p38 [50]. ERK and p38 stimulate ubiquitination and proteasomal degradation of MafA by altering its phosphorylation pattern [51], which also reduces its DNA binding. In addition to oxidizing and inactivating PKB under oxidative stress, by phosphorylation of IRS-1 serine, JNK prevents its interaction with the insulin receptor and weakens the PI3K- PKB pathway [52]. As mentioned above, the attenuation of PKB causes the activation of FOXO1 and its entry into the nucleus. Although FOXO1 has been introduced as a negative regulator of PDX1 levels, several studies such as those by Kitamura et al. [46], and Zhu et al. [19] have reported upregulation of MafA and NeuroD by FOXO1. Therefore, increase in MafA gene expression in DC group may be attributed to the activation of FOXO1, but its inability in promoting insulin gene can be attributed to change of its phosphorylation pattern by the ERK and p38. Unlike MafA, which is specific for β-cells, PDX1 is produced by both β- and non-β-cells. Although PDX1 expression is reduced by FOXO1 in β-cells, due to tissue damage caused by STZ, PDX1 is expressed in non-β- islet cells to repair tissue and differentiate into β-cells [52]. Therefore, the increase in PDX1 in the pancreas of the DC group can be attributed to non-β-cells, but cell line studies are needed. However, histopathological results showed that despite its high expression in the DC group, PDX1 is unable to perform significant tissue repair, most likely due to the severity of tissue damage caused by STZ, oxidative stress and hyperglycemia. In addition, the low levels of blood insulin in the DC group may be related to minimal functional levels of MafA and PDX1. According to histological and biochemical results, HENS treatment reduced oxidative stress and improved pancreatic tissue in diabetic rats. Consistent with the decrease in fasting blood glucose, expression of both MafA and PDX1 was decreased in HENS-treated diabetic rats.

## Conclusion

This study showed that although STZ-induced oxidative stress increased the expression levels of endogenous antioxidant system activators, namely Nrf2 and FGF21, the extent of tissue damage was greater than that this system was able to reduce TOS and MDA levels, and increase TAC. Despite the high expression level of PDX-1 and MafA genes, the high blood glucose and low blood insulin levels in rats of DC group can be attributed to FOXO1 activation under oxidative stress, which decreases the molecular activity of both factors. The reason for this statement is the reduction of oxidative stress by HENS and the adjustment of the values ​​of the above factors to be comparable to the normal control group, although this assumption requires cell line study. The histological studies in HENS-treated groups showed a reduction in tissue damage due to reduced oxidative stress by HENS. Since the rats were fasting at the time of sampling, this study demonstrated that pancreatic PDX-1 and MafA expression levels were proportional to the reduction in blood glucose levels and increase in blood insulin concentration in HENS-treated rats. By confirming the effect of HENS on transcription factors affecting insulin gene expression in this study, the next step in research will be carried out in related cell lines, taking into account the active constituents of *N. sativa*.

## Data Availability

Data presented in this manuscript is available upon request.

## Abbreviations

*N. sativa*: *Nigella sativa*
HENS: Hydroalcoholic extract of *N. sativa*
Nrf2: Nuclear factor erythroid 2-related factor 2
FGF21: Fibroblast growth factor 21
PDX1: Pancreatic and Duodenal Homeobox 1
JNK: C-Jun N-terminal kinases
FOXO1: Forkhead Box O1
